# Supplementary figures and images for: Identifying academic success and underperformance: The discriminative power of very short answer questions and multiple-choice questions
Source: PLoS One. 2026 Jul 23;21(7):e0349318. doi: 10.1371/journal.pone.0349318 (PMC13395311; doi:10.1371/journal.pone.0349318)

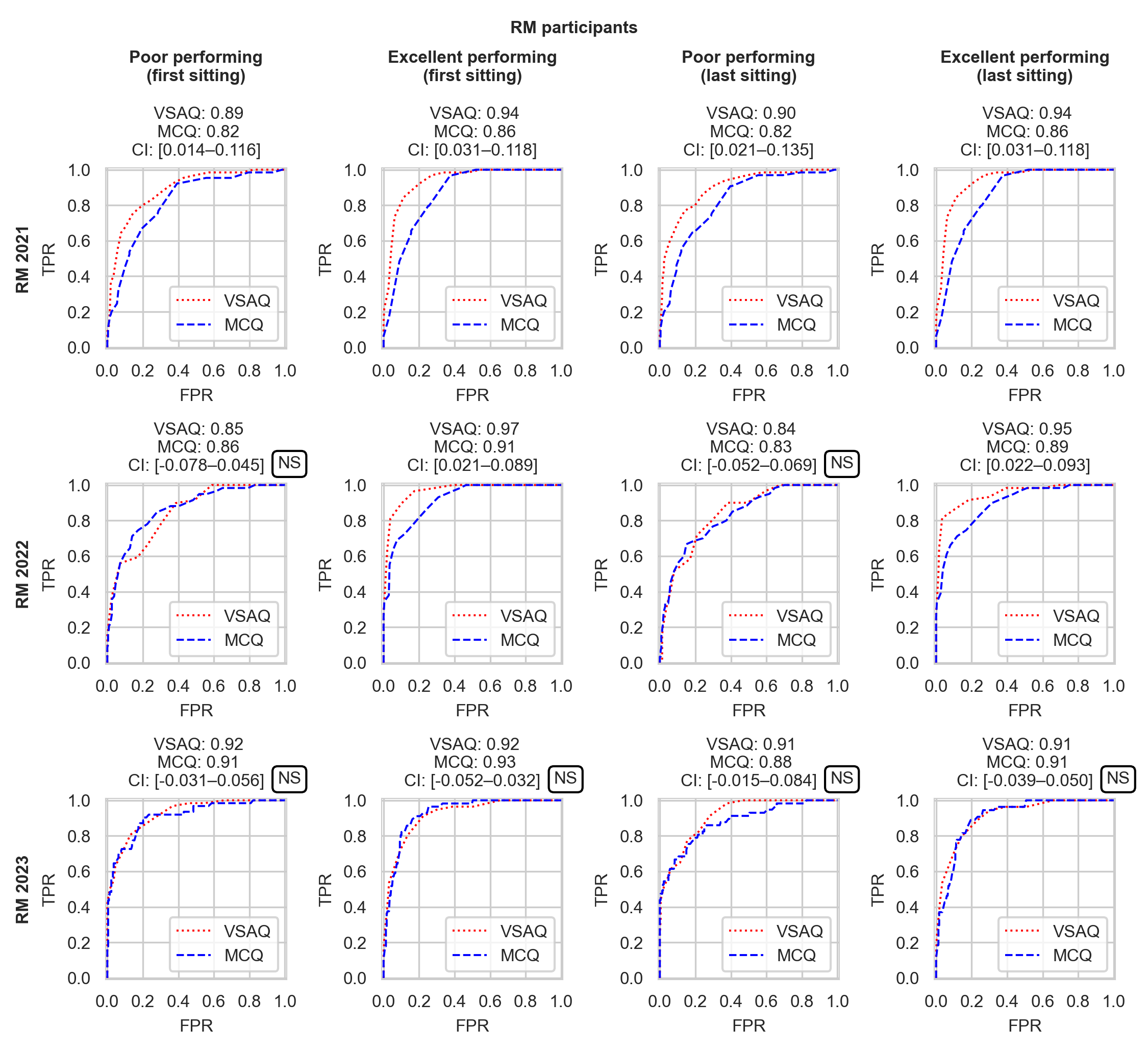

Supplement: S1 Fig — (TIFF) [file pone.0349318.s001.tiff]

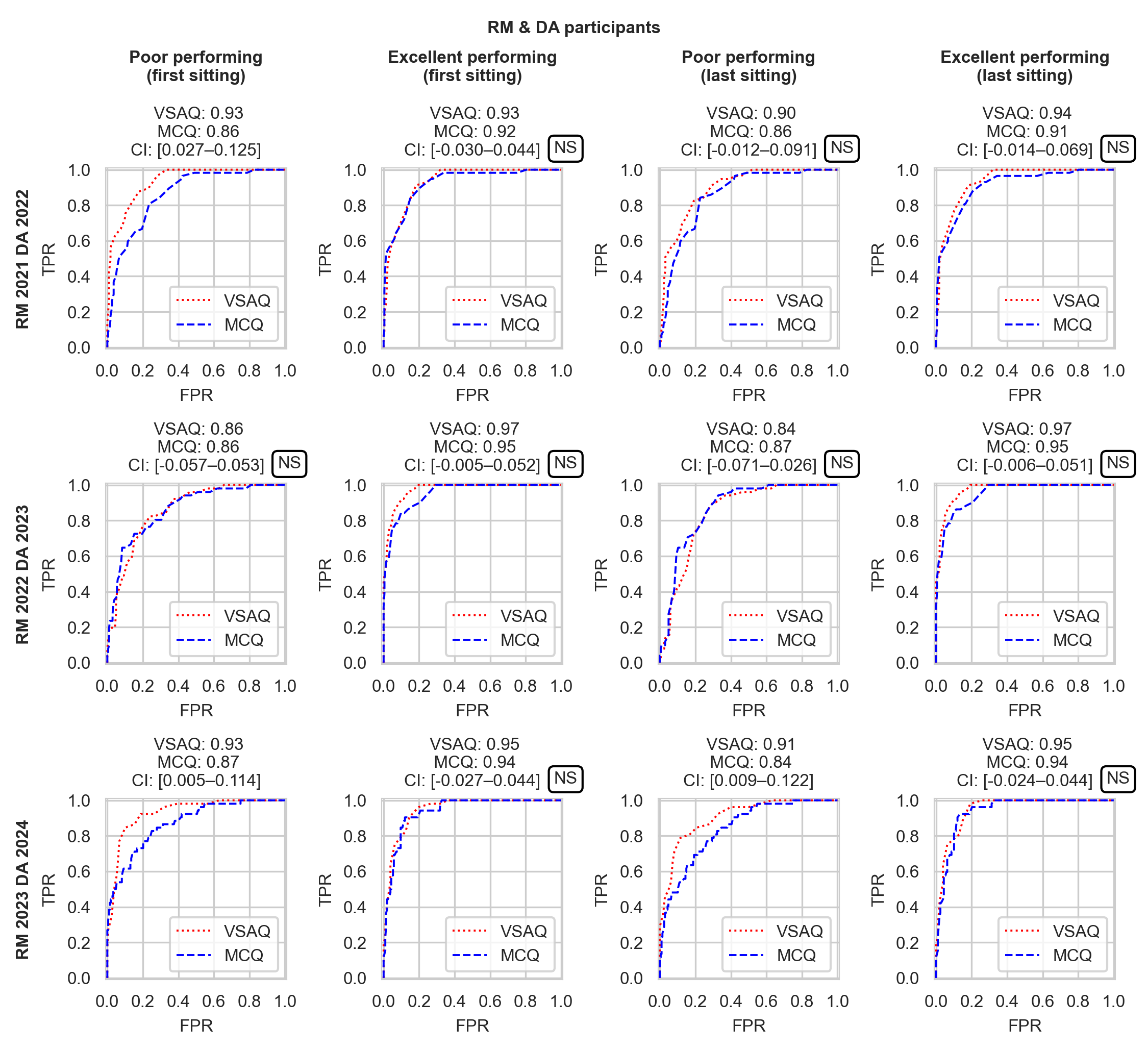

Supplement: S2 Fig — (TIFF) [file pone.0349318.s002.tiff]
