# Supplementary material for: Identifying academic success and underperformance: The discriminative power of very short answer questions and multiple-choice questions
Source: PLoS One. 2026 Jul 23;21(7):e0349318. doi: 10.1371/journal.pone.0349318 (PMC13395311; doi:10.1371/journal.pone.0349318)
Supplement: S1 Table — (PDF) [file pone.0349318.s003.pdf]

S1 Table. Courses included in the calculation of the GPA and their corresponding ECTS credits

Academic Year	Course	ECTS credits
Year 1	From Human to Cell	7.0
Year 1	From Cell to Molecule	6.0
Year 1	Academic and Scientific Development 1	2.5
Year 1	From Basic Principles to Homeostasis	8.0
Year 1	Professional Development B1	3.0
Year 1	Regulation and Metabolism	7.0
Year 1	Brain Function and Control	7.0
**Total Year 1**		**40.5**
Year 2	Pharmacology B2	2.0
Year 2	Diseases of the Abdomen	8.0
Year 2	Diseases of the Chest and Kidney	8.0
Year 2	Clinical Problems of Psychological Functioning	5.0
Year 2	Academic and Scientific Development 2	2.5
Year 2	Professional Development B2	2.5
Year 2	Mechanisms of Disease 1	7.0
Year 2	Mechanisms of Disease 2	7.0
Year 2	Clinical Problems of Movement and Musculoskeletal Function	9.0
**Total Year 2**		**51.0**
