# Supplementary material for: Identifying academic success and underperformance: The discriminative power of very short answer questions and multiple-choice questions
Source: PLoS One. 2026 Jul 23;21(7):e0349318. doi: 10.1371/journal.pone.0349318 (PMC13395311; doi:10.1371/journal.pone.0349318)
Supplement: S2 Table — (PDF) [file pone.0349318.s004.pdf]

**S2 Table.** Distribution of VSAQs and MCQs across the themes within the examinations

| | **RM '21** | | **RM '22** | | **RM '23** | | **DA '22** | | **DA '23** | | **DA '24** | |
|---|---|---|---|---|---|---|---|---|---|---|---|---|
| | VSAQ | MCQ | VSAQ | MCQ | VSAQ | MCQ | VSAQ | MCQ | VSAQ | MCQ | VSAQ | MCQ |
| Theme 1 | 2 | 1 | 1 | 1 | 1 | 3 | 2 | 4 | 4 | 3 | 4 | 1 |
| Theme 2 | 5 | 7 | 6 | 9 | 5 | 5 | 4 | 5 | 4 | 7 | 3 | 3 |
| Theme 3 | 23 | 6 | 13 | 11 | 14 | 13 | 2 | 4 | 2 | 5 | 4 | 1 |
| Theme 4 | 4 | 11 | 5 | 8 | 5 | 10 | 3 | 4 | 6 | 3 | 7 | 1 |
| Theme 5 | 5 | 6 | 3 | 6 | 4 | 6 | 3 | 8 | 2 | 7 | 4 | 2 |
| Theme 6 | 3 | 1 | 1 | 1 | 2 | 1 | 2 | 11 | 6 | 4 | 5 | 4 |
| Theme 7 | *NA* | *NA* | *NA* | *NA* | *NA* | *NA* | 5 | 7 | 3 | 5 | 1 | 4 |
| Theme 8 | *NA* | *NA* | *NA* | *NA* | *NA* | *NA* | 1 | 5 | 3 | 6 | 3 | 4 |

*RM=Regulation and Metabolism; DA=Diseases of the Abdomen; VSAQ=very short answer question; MCQ=multiple-choice question.*

*RM: Theme 1: Regulation of the temperature, Theme 2: Regulation of the reproduction, Theme 3: Regulation of the thyroid, Theme 4: Stomach, bowl and liver, Theme 5: Metabolism, Theme 6: Nutrition.*

*DA: Theme 1: Abdominal swelling, Theme 2: Stomach complaints, Theme 3: Jaundice, Theme 4: Acute abdominal pain, Theme 5: Chronic abdominal pain and defecation disorders, Theme 6: Blood loss, Theme 7: Anatomy, Theme 8: Other.*
